# Xeroderma Pigmentosum: General Aspects and Management

**DOI:** 10.3390/jpm11111146

**Published:** 2021-11-04

**Authors:** Monica Piccione, Anna Belloni Fortina, Giulia Ferri, Gloria Andolina, Lorenzo Beretta, Andrea Cividini, Emanuele De Marni, Francesca Caroppo, Ugo Citernesi, Rosa Di Liddo

**Affiliations:** 1 Department of Pharmaceutical and Pharmacological Sciences, University of Padova, 35131 Padova, Italy; 2 Pediatric Dermatology Unit, Department of Medicine DIMED, University of Padova, 35128 Padova, Italy; anna.bellonifortina@unipd.it (A.B.F.); francesca.caroppo@outlook.it (F.C.); 3 I.R.A. Istituto Ricerche Applicate S.p.A., 20865 Usmate Velate, Italy; giulia.ferri@iralab.it (G.F.); gloria.andolina@iralab.it (G.A.); lorenzo.beretta@iralab.it (L.B.); andrea.cividini@iralab.it (A.C.); emanuele.demarni@iralab.it (E.D.M.); citernesi@iralab.it (U.C.)

**Keywords:** xeroderma pigmentosum, nucleotide excision repair, personalized medicine, liposomes, Dimericine

## Abstract

Xeroderma Pigmentosum (XP) is a rare genetic syndrome with a defective DNA nucleotide excision repair. It is characterized by (i) an extreme sensitivity to ultraviolet (UV)-induced damages in the skin and eyes; (ii) high risk to develop multiple skin tumours; and (iii) neurologic alterations in the most severe form. To date, the management of XP patients consists of (i) early diagnosis; (ii) a long-life protection from ultraviolet radiation, including avoidance of unnecessary UV exposure, wearing UV blocking clothing, and use of topical sunscreens; and (iii) surgical resections of skin cancers. No curative treatment is available at present. Thus, in the last decade, in order to prevent or delay the progression of the clinical signs of XP, numerous strategies have been proposed and tested, in some cases, with adverse effects. The present review provides an overview of the molecular mechanisms featuring the development of XP and highlights both advantages and disadvantages of the clinical approaches developed throughout the years. The intention of the authors is to sensitize scientists to the crucial aspects of the pathology that could be differently targeted. In this context, the exploration of the process underlining the conception of liposomal nanocarriers is reported to focus the attention on the potentialities of liposomal technology to optimize the administration of chemoprotective agents in XP patients.

## 1. Introduction

In the last decades, nanoparticle-mediated drug delivery has been considered as a successful approach in numerous medical fields, including dermatology. In particular, rare skin diseases, such as Xeroderma Pigmentosum (XP), have been considered for the application of liposomal nanocarriers of the DNA repair T4 endonuclease 5 (T4N5) enzyme. Both in vitro and in vivo studies have demonstrated high efficiency to minimize UV-induced photolesions with low-adverse effects in comparison to the most popular XP treatments. Although this approach positively passed pre-clinical trials of phase I and II, to date, phase III studies have been not yet successfully concluded, highlighting pros and cons of T4N5 liposomal lotion in XP patients.

The present review has been designed to explore the molecular basis of XP and to present the history of the therapeutic approaches proposed as sunlight protection systems in healthy and sensitive people, such as XP patients. This knowledge, together with the understanding of the long and difficult process that led to the design of this liposomal-mediated approach—and its relative limits—could be essential for scientists to take further steps in this challenging field.

### Clinical Features of Xeroderma Pigmentosum

Xeroderma Pigmentosum (XP) is a rare heritable disorder with 100% penetrance, autosomal recessive, characterized by an enzymatic defect in the DNA-repair pathway known as nucleotide excision repair (NER) [1,2]. The grade of incidence of XP is dependent on geographic distribution (2.3/1,000,000 births in Western Europe), and males and females are equally affected [2,3,4].

XP patients commonly show specific clinical signs including an extreme sensitivity to UV exposure (with consequent painful sunburns), skin dryness (also called xerosis), progressive pigmentary abnormalities freckle-like (as suggested from the term “pigmentosum”), different levels of skin damage, early photoaging, and an increased incidence of face/head/neck malignant tumours. Moreover, some patients show progressive neurologic and ophthalmologic degeneration [2,3,5,6]. Compared to the health population, XP patients demonstrated a skin cancer occurrence that is increased up to 10,000-fold for non-melanoma skin cancer (NMSC), such as basal cell carcinoma (BCC) and squamous cell carcinoma (SCC), while melanoma is reported to be 2000-fold more frequent in xeroderma pigmentosum [7,8].

High heterogeneity is observed in XP symptomatology and is strictly associated to the genetic alteration that is responsible of the pathology onset. The international guidelines establish that the diagnosis of XP must be formulated following the identification of biallelic mutation in eight different genes, listed in Table 1. Based on clinical findings and family history, XP patients are classified in seven complementation groups (from XP-A to XP-G), together with a XP variant (XP-V), consisting of a condition with a functional NER but a lacking DNA polymerase (Pol η), that is involved in the replication of damaged DNA [6,9]. Due to the involvement of each gene in a specific step of NER process, all complementation groups show wide variable and characteristic clinical manifestations (Table 2), with severity grade and age of onset partially dependent on environmental factors, such as the exposure to sunlight [9,10]. The clinical signs common to all XP groups are the onset of skin pigmentary alterations and an impaired sensitivity to sun exposure, that is exacerbated on time by xerosis. Conversely, lesions at Central Nervous System (CNS) and eyes or invasive skin cancers occur only in particular groups, showing a severe gravity score [11].

**Table 1:** Loci and genes associated with XP complementation groups and XP variant.

Group	Gene Involved [4]	Locus [4]	Frequency (%) [6,12]
XP-A	*XPA*	9q22.33	30
XP-B	*ERCC3/XPB*	2q14.3	0.5
XP-C	*XPC*	3p25.1	27
XP-D	*ERCC2/XPD*	19q13.32	15
XP-E	*DDB2/XPE*	11p11.2	1
XP-F	*ERCC4/XPF*	16p13.12	2
XP-G	*XPG*	13q33.1	1
XP-V	*DNApol η*	6p21.1	23.5%

**Table 2:** Clinical signs of Xeroderma Pigmentosum based on complementation groups. (M) mild; (S) severe; (+) symptomatic manifestation; (−) absence of symptoms.

Group	Gravity Score [11]	Photosensitivity (Sunburn)	Xerosis	Pigmentary Abnormalities	Increased Skin Cancer Risk	Neurological Disorders	Eye-Disorders
XP-A	M/S	+/−[13]	+[14]	+[13]	+[11]	+[15]	+[14]
XP-B	M	+[16]	+[16]	+[16]	+[11,16,17]	+[6,18,19]	+[19]
XP-C	M/S	+[20]	+[20,21]	+[20,21]	+[11,20,22,23]	−[6,9,20,24]	+[20]
XP-D	M	+[25]	+[26]	+[25]	+[11]	+[6,18,27]	+[6]
XP-E	M	+[2]	+[2]	+[2]	+/−[11,28]	−[6]	+[6]
XP-F	V	+[29]	+[29]	+[29]	+[11]	−[6,30]	+[6]
XP-G	M/S	+[26,31]	+[26]	+[2]	+[11,32]	+[6,18,31]	+[6]
XP-V	V	+/−[10,18]	+[33]	+/−[18]	−[11]	−[6]	+[6,34]

**Figure 1:**
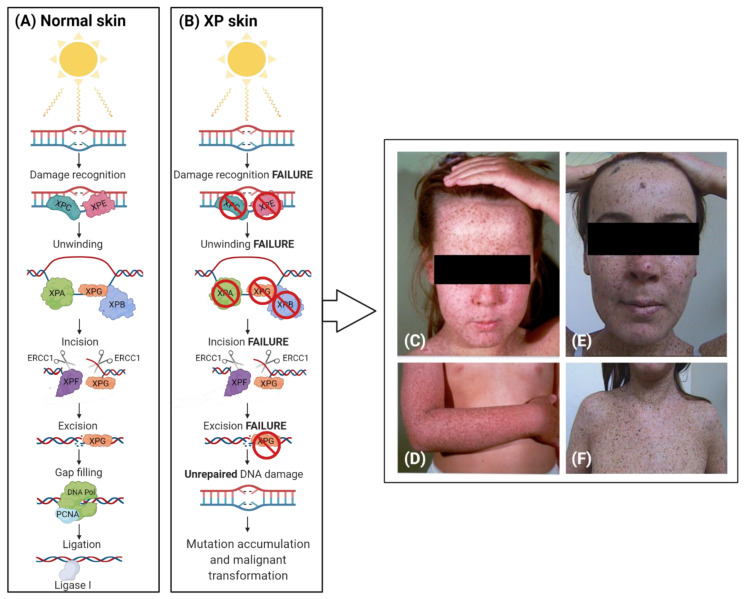
Schematic representation of DNA repair machinery in normal (**A**) and XP (**B**) skin. (**C**–**F**) Some clinical features of UV-induced damage in XP patients are reported. (Created with BioRender.com (accessed on 10 September 2021)).

From the histological point of view, the skin of XP patients is characterized by massive structural and functional alterations due to the failure in the removal of UV-induced lesions. Numerous studies extensively reported that UV exposure is the primary exogenous factor involved into the disruption of the epidermal complex architecture [35,36,37]. In physiological conditions, more than 70% of melanomas are caused by UV because of its involvement in the modulation of cell growth/differentiation, and tumour initiation/promotion [37]. Among the three groups of UV rays (UVA, UVB, and UVC), UVA (315–400 nm) has been demonstrated to infiltrate into the epidermis and dermis, triggering the production of oxidative free radicals. UVB (280–315 nm) penetrates less deep into the dermis, where, on the other hand, induces DNA photolesions and acts as inflammatory stimulus (Figure 1) [38]. Due to the light scattering/absorption/reflection, these direct effects cause the photoaging phenomenon, which is highly exacerbated in XP patients [39].

The histological analysis of XP skin biopsies often displays atrophy, hyperkeratosis, consisting of epidermal depressions filled with keratin fibres, together with impaired immune cell infiltrates (i.e., lymphocytes and eosinophils) into the dermis, irregular areas of abundant melanin-containing pigment cells, an increased number of melanocytes in the basal lamina and capillary expansion [40]. Moreover, the main feature observed in the bioptic samples of XP patients is a typical dysplasia [41].

## 2. The Complex Involvement of NER

In physiological conditions, mammalial cells counteract the onset of UV-induced DNA lesions through different complex machineries, each of them is responsible for the repair of a wide range of genome alterations. Taken together, these events determine an elaborate cellular response which could be synthetized in different phases: (i) cell cycle arrest; (ii) DNA repair; (iii) reprise of cell cycle (when the repair was efficient) or apoptosis (essential whether the repair pathways were ineffective) [42]. The main goal of these mechanisms is to minimize the accumulation of mutations potentially tumorigenic.

The cellular response to DNA damage is strictly dependent on UV intensity: low doses lead to the inhibition of DNA replication and the transient arrest of cell cycle, while high doses directly trigger a replicative arrest followed by p53- and Bcl2- mediated apoptosis [43]. Based on DNA damage, five categories of DNA repair pathways can be triggered. Among these, the nucleotide excision repair is responsible for the removal of the damaged base(s) through a double break of the phosphodiester bonds in the DNA strand at determined number of nucleotides from the damage. Subsequently, enzymatic activities contribute to synthetize and ligate oligonucleotides into the strand-gap [44].

**Figure 2:**
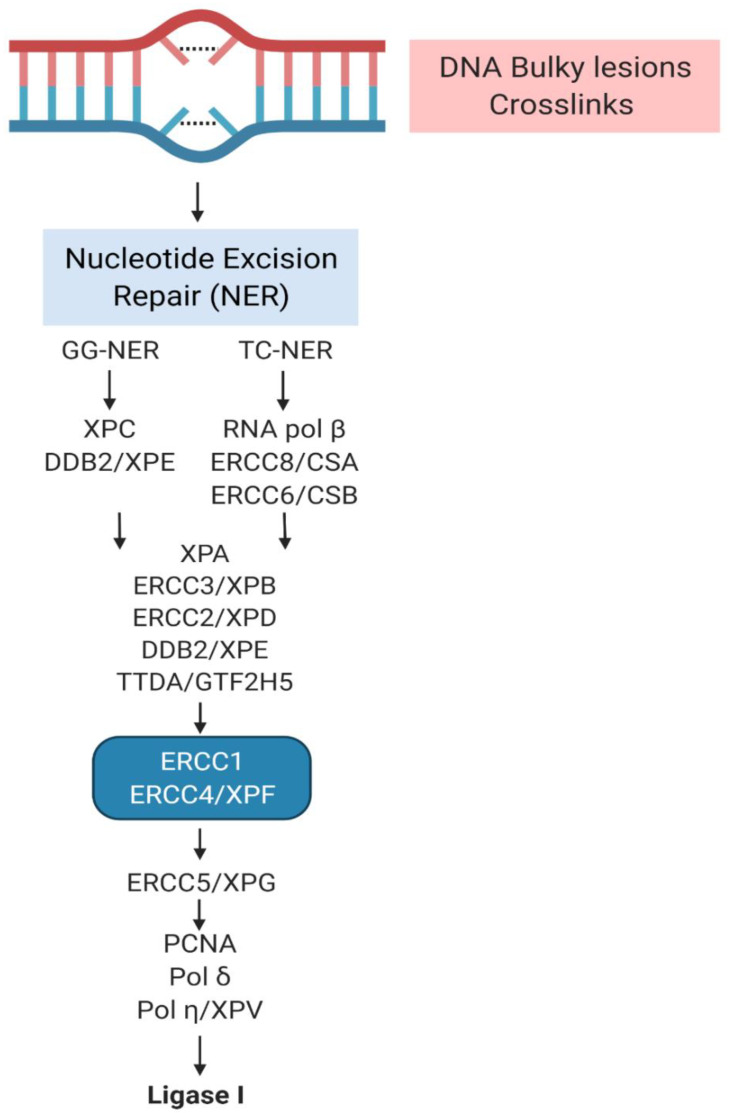
Schematic of the nucleotide excision repair (NER) pathway in humans (created with BioRender.com (accessed on 10 September 2021)). Mutations that occur in genes encoding for the proteins of this DNA-repair system are responsible for the onset of Xeroderma Pigmentosum.

NER is one of the main pathways involved in the removal of UVB-induced DNA photolesions, and its efficiency ensures a significant protection against mutations and cancer induction in the skin after sun exposure (Figure 2) [3]. The most important feature of NER is the ability to recognize an extremely wide range of photolesions, including UV-induced cyclopyrimidine dimers (CPDs) and 6–4 photoproducts (6-4PPs) [45,46].

In mammals, the NER machinery consists of two distinct pathways, which act depending on the recognition system of the nucleotide damage: the transcription-coupled NER damage recognition (TC-NER) triggers the rapid removal of base lesions from transcribed strands, whereas the global genome NER (GG-NER) is generally slower and detects bulky lesions anywhere in the genome promoting the repair of DNA independently of transcription [47,48], throughout the entire cell cycle [45,49,50]. 

The GG-NER is promoted by the activation of the GG-NER specific factor XPC, in some cases associated with the damaged DNA-binding protein 2 (DDB2)/XPE. Conversely, TC-NER is initiated by RNA polymerase β, together with TC-NER specific factors ERCC8/CSA and ERCC6/CSB [51,52].

After the recognition of DNA damage, the transcription factor IIH (TFIIH) complex is recruited. TFIIIH is a multifunctional protein whose composition is variable. It is composed of seven subunits, including ERCC3/XPB translocase and ERCC2/XPD helicase, or ten parts. TFIIH function is to bind the DNA lesion and to unwind the DNA duplex around the lesion, in order to facilitate the assembly of the repair machine. Subsequently, the repair factors XPA and ERCC5/XPG are recruited and associate to the TFIIH core. Both XPA and XPG induce a conformational stabilization of TFIIH, necessary for the helicase activity of XPD. Moreover, XPA contributes to the damage verification through its bond to the 5′ side of the DNA damage [51]. When the double strand is denatured, the repair bubble binds to the replication protein A (RPA), that protects the undamaged strand from nuclease attack [51,52,53,54]. The XPA recruits the NER pre-incision complex: the first incision is performed by the endonucleases ERCC4/ XPF–ERCC1 from the 5′ side to the damage site, generating a free 3′-OH end; the XPG makes a second incision that exposes a free 5′-phosphate group [55,56]. The oligonucleotide that contains the lesion is released from the repair bubble in complex with TFIIH. Then, after the dissociation of TFIIH from the DNA, the oligonucleotide is bound by RPA or degraded by cellular nucleases [57].

Subsequently, the replication machinery is activated. The synthesis can be performed through different systems, which include the proliferating cell nuclear agent (PCNA) and DNA polymerases δ or η. The free 5′-phosphate end is utilized in the final step of NER, and is catalysed by DNA ligase I or DNA ligase III [44,58,59].

Mutations in any of the seven genes encoding for the proteins involved in NER result into the abnormal repair of DNA, as usually observed in XP patients [5,47]. As a consequence, the cells progressively accumulate the DNA photoproducts, which are potentially tumorigenic.

As previously reported, CPDs are the most represented photoproducts, while the 6-4PPs amount corresponds to the 25–30% of total CPDs. The DNA destabilization caused by UV-induced lesions is directly related to the repair efficiency: CPDs induce a minimal distortion of the double helix; conversely, the 6-4PPs cause a DNA backbone bending. Hence, CPDs are commonly excised with a slower kinetic [59].

Due to the pivotal role exerted by NER machinery into the onset of XP, new-generation approaches considered this pathway as an ideal therapeutic target.

## 3. XP Molecular Insights

**Figure 3:**
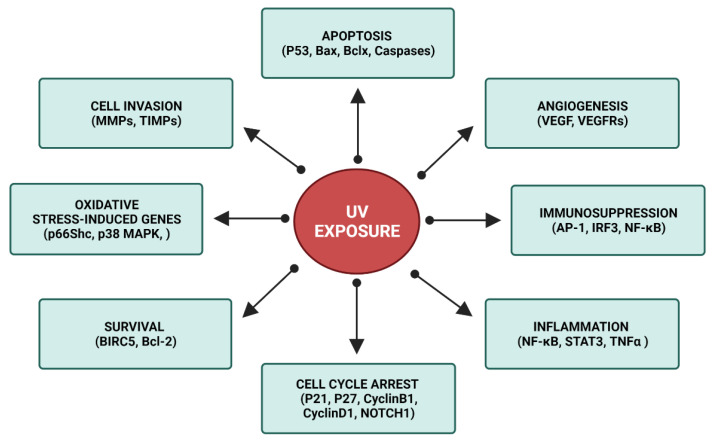
Schematic representation of the intracellular pathways triggered by UV exposure (created with BioRender.com (accessed on 10 September 2021)).

The increased susceptibility to UV radiation in XP patients is strictly related to their failure to counteract via NER the formation of photoproducts in DNA [39]. The complex symptomatologic picture observed in XP patients reflects the physiological response to UV exposure, triggered by the photolesion of biomolecules and the consequent activation of numerous intracellular pathways (Figure 3) [9,39,60,61,62,63,64,65,66,67,68,69,70].

Photons are highly reactive species that directly or indirectly interact with a wide variety of cellular components, including phospholipids, DNA and proteins [71]. Direct effects occur when biomolecules are chemically modified by UV absorption; the indirect interactions result from the interplay between UV-induced Reactive Oxygen Species (ROS) and cellular components [72]. Among the most targeted biomolecules, DNA absorbs the energy of photons causing the formation of a large variety of photoproducts [73].

The interaction between UVB and DNA triggers the formation of cyclobutane pyrimidine dimers (CPDs) and/or pyrimidine (6-4) pyrimidone photoproducts ((6-4) PPs). Among DNA photoproducts, CPDs demonstrate to exert the highest mutagenic potential in mammalian cells because of their ability to rapidly repair only (6-4) PPs [74,75]. Therefore, CPDs persist longer in skin tissue, leading to DNA polymerase errors due to the helix distortion and the accumulation of mutations [76].

The formation of photoproducts occurs via a very rapid mechanism: most CPDs are generated within picosecond post-UV exposure, although Premi et al. (2015) [77] demonstrated that in some skin cell types (i.e., melanocytes), this process is prolonged and delayed (up to 3 h) as a consequence of further chemical reactions. Thus, the first few hours post UV exposure could be considered as a therapeutic window to restrict the formation of photoproducts [66].

The biological effects of CPDs formation could be classified, depending on the timing of sun exposure, as transient, acute, or chronic. Vink et al. (2001) [78] reported that transient effects include the direct or indirect affection of the immune response. In particular, cutaneous immune cells are targeted by UV, which also leads to an immunosuppressive environment through the regulation of cytokine’s synthesis. Acute effects are instead triggered by the cytokine/growth factors receptors in epidermal and dermal cells, primed by increased oxidative stress. In particular, tumour necrosis factor (TNF)α, interleukin (IL)1β, and IL6 are produced after UV exposure and exert an essential role in the progress of skin inflammatory processes [79]. Additionally, the activation of nuclear factor kappa-light-chain-enhancer of activated B cells (NF-kB) pathway, mediated by ROS, contributes to exacerbate the cutaneous acute symptoms [80,81,82]. The inflammatory process is macroscopically evident through the dilatation of dermal blood vessels and skin redness (also known as sunburn/erythema).

Moreover, cells are able to counteract the injury through the activation of a series of checkpoints, whose function is to delay the cell cycle progression in order to achieve the time necessary to restore the genomic damage [48]. Moreover, DNA damage leads to the activation of apoptotic signalling cascades through specific transcription factors (e.g., p53, c-FOS, NF-κB) [83,84,85]. The generation of photoproducts is responsible of chronic skin damages, that includes also skin cancers. All types of UV-induced DNA lesions trigger the development of precancerous lesions, depending on the targeted nucleotide sequence [86,87]. When DNA repair is deficient (i.e., XP), this mutagenic process is accelerated, leading to an anomalous formation of fibrous tissue—and consequent premature skin ageing—and to the onset of skin cancers in childhood [20,78,88].

The peroxidation of membrane lipids also causes an altered membrane permeability and fluidity, while protein oxidation promotes an altered protein activity and function, as reported by Davies (2016) [68,89,90].

The complex process underlying the UV-induced skin damage is further exacerbated by the depletion of antioxidant molecules such as glutathione, that guarantees a protection against free radicals [91].

Moreover, the nuclear translocation of the activator protein 1 (AP-1) simultaneously silences the synthesis of de novo pro-collagen type I and III and induces the activation of matrix metalloproteinases (MMPs) that degrade mature collagen fibres [81,82]. As a consequence of this process, XP patients show an improved demolition of extracellular fibres in dermis and a dysfunctional turnover of extracellular matrix (ECM) [88].

In addition to the mechanisms counteracting UV-induced damages, XP patients present altered levels of ROS in the skin cells. Most of UVA-induced damage to biomolecules (DNA and proteins) is induced indirectly via interaction with intracellular photosensitisers that, in turn, trigger the generation of reactive oxygen species [43].

Epidermal cells are vulnerable to ROS, that, after an excessive sun exposure and an unbalanced equilibrium between oxidative stress and antioxidants, could initiate or maintain the malignant transformation of cutaneous cells. ROS are physiologically produced in the human cells by mitochondria, but their synthesis can also be triggered by UV, which also induces the depletion of the anti-oxidant catalase [88].

As accurately described by Polefka et al. (2012) [89], the radiation dose and the duration of sun exposure are crucial to determine uncontrolled biological effects [37,39].

All the molecular mechanisms underlying the malignant transformation of cutaneous cells after UV exposure have been suggested in past years as targets for numerous therapeutic approaches. 

## 4. First-Generation Intervention Strategies

To date, the management of XP patients follows international guidelines and consists in early genetic/clinical diagnosis and a long-life protection from UV exposure. The reduction of sunlight exposure is performed through the daily application of total sunscreen creams or wearing gloves, hats, sunglasses, using glasses that exclude UV rays on windows into the car, and avoiding the exposure during the day [92,93].

There are no curative treatments for XP, thus the cancer-free survival of patients is based on permanent full body protection where possible and frequent surgical resection of skin cancers.

### 4.1. Physical/Chemical Photoprotectors

According to FDA guidance and regulatory information, numerous sunscreens have been produced and commercialized to enhance the ultraviolet protection of XP patients in terms of UVA/UVB coverage, sun protection factor (SPF), water resistance, and effective duration of shielding [94].

Among the topical sunscreen agents, physical and chemical sun lotions are the most recommended products. Physical blockers (also called inorganic filters) act as a protective screen of skin, while chemical absorbers (also called organic filters) soak up UVA and UVB radiation. Moreover, physical sunscreens contain elements such as titanium dioxide, zinc oxide, red ferric oxide, talc, nanoparticles (NPs), and kaolin, that exert their functions through the dispersion and the reflection of UV, infrared rays (IRRs), and visible light [95]. Chemical sun lotions work by absorbing UV radiation and often contain a combination of ingredients to provide a coverage against UVB or UVA or against both of them. For example, aminobenzoates selectively absorb UVB, while benzophenones are able to selectively absorb UVA. Chemical sunscreens are efficient to full protect skin from UVB; but not from UVA [96].

In order to obtain a daily full protection from UV, XP patients should observe a strict treatment programme, that is dependent on the average time of shielding exerted by the commercial sunscreens. Dermatologists recommend a first total morning coverage, followed by reapplications every 2/3 hours over the skin areas exposed to UV, using up to 30 SPF and a sunscreen that includes UVA and UVB blockers. Moreover, the cost, the ease of application and the skin comfort after application are conditions that clinicians take into consideration to define a sunscreen as suitable for XP patients [97,98].

It is known that sun lotions guarantee only a partial protection and could induce toxicity with a frequent use [95,99]. As reported by Jansen et al. (2013) [100], oxybenzone induces a low rate of photo-allergenicity and is harmful for the environment (i.e. bleaching of coral reefs) [101].

An intrinsic toxicity has been demonstrated for the nanoparticles (NPs) derived from titanium dioxide and zinc oxide. The application of sunscreens containing NPs could lead to their deep absorption and consequent aggregation, causing cytotoxicity and genotoxicity [102]. New formulations in which NPs are coated with organic or inorganic compounds have been produced in order to avoid these adverse effects [103]. Wright et al. (2001) [104] demonstrated that coated NPs do not stimulate Langerhans cells and inflammatory response.

Nevertheless, some molecules and their derivatives in sunscreens (i.e., salicylic acid esters and benzophenones) are reported to reach the dermis and to induce allergic reactions [105]. Even oxybenzone has been included in the list of photoallergens by the European Scientific Committee on Consumer Safety in 2006 [106]. Moreover, as these substances have been also found in urine, they have been suspected to be absorbed by the circulatory system and to have potential systemic effects. Thus, for their use, the concentration of benzophenones in sunscreens has to be in accordance with FDA guidelines [103,107].

### 4.2. Dermabrasion and Chemical Peeling

Among the alternative approaches initially considered to treat XP patients there was the dermatome shaving, also known as dermabrasion [108]. It is a resurfacing technique suggested for the treatment of scars, wrinkles, and photodamage with a moderate effectiveness and safety [109,110].

Ocampo et al. (1996) [111] proposed this method to treat a 2-year old female affected by XP. When the patient developed actinic keratoses (AK) and numerous SCCs/BCCs at 17 months of age, skin cancers have been removed by surgical procedures and dermabrasion has been performed using an abrasive diamond fraise. After a postoperative care, in combination with other treatments, the patient showed a significantly reduced probability to develop new tumours, without any delay in reepithelization [111].

Despite its benefits, dermabrasion does not counteract the onset of further actinic damage in XP patients. Moreover, its clinical use has been replaced by newer techniques such as chemical exfoliation. To date, dermabrasion is still a useful therapeutic option for scar revision from acne or traumatic scars [109].

The chemical peeling has substituted the older dermabrasion for the treatment of xeroderma pigmentosum as the multiple interventions required for XP patients are impracticable in dermabrasion but more feasible in chemical peeling [112,113].

Up to now, the trichloroacetic acid (TCA) peel is considered as the gold standard of chemical peeling [112]. Overall, the chemical agent is carefully chosen based on the depth of penetration required. This feature defines the classification of chemical peels in superficial (that exfoliate the epidermal layer), medium (that achieve the upper layer of dermis), and deep (that include also the papillary dermis). The trichloroacetic acid (TCA) is considered as a medium peeling agent, due to its capability to penetrate into the skin, reaching the most external dermis [114]. The TCA induces a superficial coagulation of skin proteins and a consequent degradation of the epidermis and the upper dermis. This chemical reaction leads to the activation of regenerative processes that trigger the renewal of damaged skin. When applied on XP patients, chemical peeling demonstrated to make the skin smooth, reducing the dryness and the inflammatory response. This suggested a higher grade of treatment-acceptance by patients. Moreover, it showed to guarantee a tumour-free period of 2–5 years. As for the dermabrasion, the efficacy of the chemical peeling is only transient, but it is a less complex method to reduce the onset of post-treatment diseases (i.e., infections) [112,115].

### 4.3. Administration of Retinoids

The widespread need to identify an efficient therapy to contol XP symptoms triggered researchers to explore other therapies, such as the topical administration of retinoic acid (vitamin A). This approach exerted a protective role against the onset of skin neoplasms in XP patients, as demonstrated by Bollag et al. (1970) [116]. Further studies evaluated the side effects of topical retinoids, highlighting their susceptibility to UV, with consequent dose-dependent erythema, burning, and skin irritation [41,117,118]. Thus, second and third generation retinoids (vitamin A derivatives) were considered for a systemic treatment of XP. The most common retinoids suggested by dermatologists are the etretinate, the acitretin, and the isotretinoin [119]. These molecules have demonstrated to exert a protective role against the development of cutaneous precancerous lesions. In particular, the oral administration of isotretinoin (Roaccutane) is considered as the most effective to prevent BCC and SCC, due to its control on the proliferation of keratinocytes [7,120,121].

Nowadays, despite the recognized protective effects, numerous adverse reactions to retinoids have been documented. Among these, pharmacological (i.e., cheilitis, eye/nasal dryness, conjunctivitis, epistaxis, and irritant dermatitis) and toxic (i.e., high hepatic enzymes, increased levels of triglycerides and cholesterol) effects have been reported [122,123]. In some patients, isotretinoin has shown to induce teratogenicity and skeletal abnormalities. Moreover, very rare side effects include headaches, joint pain, leukopenia, anaemia and an increased risk of inflammatory bowel disease (IBD) [33,119].

The possible occurrence of these effects has induced clinicians to discard this approach, in favour of other strategies.

### 4.4. Phototherapy and Laser Resurfacing

To block the onset of melanoma and non-melanoma skin cancers, techniques, such as the photodynamic therapy (PDT) and the full-face resurfacing, have been considered as useful for the treatment of xeroderma. Numerous studies have evaluated the short-term response to PDT in XP patients, describing promising results [33]. In particular, PDT’s protective activity is due to a photosensitizing agent. Under artificial light exposure, this molecule has been demonstrated to be activated and to exert anti-cancer effects [124]. 

To date, among the photosensitive molecules, the photofrin is not recommended for the treatment of XP patients because it induces excessive wound healing and consequential scarring [125]. Therefore, the topical application of 5-aminolevulinic acid (ALA)—a synthetic precursor of the protoporphyrin IX—is now indicated as the best approach. The anti-cancer effects of this treatment have been studied in several XP patients, while Procianoy et al. (2006) [126] demonstrated that PDT in XP-A patients could increase the incidence of SCC [127,128,129]. However, the exposure of XP patients to some artificial lights remains a controversial issue for dermatologists.

As for PDT, laser and carbon dioxide (CO_2_) are widely recognized as efficient techniques to prevent skin cancer in high-risk population [130,131,132,133]. The CO_2_ laser resurfacing has been considered for many years as the gold standard for the treatment of skin precancerous lesions because of its important efficacy in the remodelling of dermal collagen [134,135].

Nevertheless, it implies the onset of adverse effects, such as inflammation, scarring, altered pigmentation and a prolonged healing period [125,130]. Therefore, the fractionated/pulsed lasers are today the preferred method. This approach is the most recommended one for the treatment of actinic keratosis, because it allows the simultaneous treatment of different areas of the skin in a single intervention and leads to a rapid reepithelization (within 10–14 days) [131]. Moreover, it decreases tissue alteration grade and minimizes bleeding and scarring [133].

Both techniques are based on the absorption rate of the energy derived from the laser, that induces a fast heating of the irradiated area, with the consequent vaporization of intracellular water, inducing the ablation of tumours [131].

Laser resurfacing allows to avoid surgical treatments of precancerous lesions and is efficient to reduce the incidence of skin cancers. It also ensures long-term effects, with prolonged cancer-free intervals [136,137]. This body of evidence makes the laser technique the most selected option in XP patients for the removal of damaged skin areas for a limited period as it has been reported to be ineffective to reduce sun sensitivity and proneness to skin cancer, that is typically observed in XP patients. 

### 4.5. Chemotherapeutic Drugs

Some classes of anti-cancer drugs have been considered for the treatment of XP patients as alternative to the surgical resection of skin tumours. Among these, the topical application of 5-Fluorouracil (5-FU) or Imiquimod has demonstrated to be the most efficient drug [125]. In particular, 5-FU has shown to exert its functions through the inhibition of deoxythymidine mono-phosphate (dTMP), a key component in the replication and transcription of DNA [138,139]. Due to its involvement in fundamental cellular processes, 5-FU blockage induces p53-mediated apoptosis [138]. Numerous studies have demonstrated that the administration of topical 5-FU to XP patients is useful to negatively control the malignant transformation of superficial skin cells and to control actinic keratoses [125,139].

Despite its anti-cancer activity, 5-Fluorouracil is a non-specific drug that promotes adverse effects such as the induced cell death of non-cancerous cells and the pyroptosis, a process recently described that involves apoptosis exacerbated by inflammation. Moreover, recent studies reported that the topical treatment with 5-FU failed into the destruction of extended skin cancers, also causing painful lesions [33]. 

Similar results were obtained after a topical therapy using Imiquimod, that exerts its immunomodulator activity acting as an agonist of the Toll-like receptor (TLR) 7. The stimulation of TRL7 induces the activation of the cytotoxic immune response mediated by both cytokines (i.e., interferon-α and interleukin-12) and immune cells, such as monocytes-macrophages [140]. In the past years, the topical administration of 5% Imiquimod has been considered as an efficient tool to counteract actinic keratosis with a low recurrence rate in the following months [141,142,143,144].

As described for 5-FU, Imiquimod has shown to induce mild–severe adverse effects, particularly in paediatric patients, which are the main target of XP treatments [145,146,147].

Due to the low tolerance of side effects, together with the availability of better-endured alternative treatments (i.e., the cryogenic resection using liquid nitrogen), to date, the topical therapy using anti-cancer molecules is restricted to selected XP patients.

## 5. Next Generation Approaches: Target Therapies

Despite to their numerous benefits, all the first-generation approaches described present important side effects. Moreover, they were only aimed to increase XP patients’ quality of life and self-acceptance, without interfering with molecular processes underlying the pathology, i.e., the correction of the DNA photolesions. Thus, in parallel to the development of new therapeutic techniques and the spread of the personalized medicine concept, numerous studies have been performed to test different approaches that directly target the intracellular pathways that are responsible of malignant transformation in XP, such as NER.

### 5.1. Gene Therapy and Autologous Transplantation

In the 1990s, a wide range of genetic diseases were considered to be potentially treatable using gene therapy strategies. The possibility to modify cells—lacking or mutated for a specific gene—through the artificial introduction of the correct DNA sequence opened a large opportunity for the treatment of XP. Moreover, the simplicity to access to the skin, together with its large working-surface caused the speculation that the skin could be an optimal target for gene delivery. As reported by Carreau et al. (1995) [148], numerous studies successfully demonstrated that the recovery of the DNA repair machinery was feasible in skin fibroblasts after a stable or transient insertion of the functional gene that was deficient in a specific complementation group.

In numerous gene therapy protocols, modified retroviral viruses (deprived of their pathogenic potential) were used as vectors because of their high capability to carry genetic material into cells obtained from XP patients [148,149,150]. Fenjives et al. (1994) [151] tested the gene therapy targeting epidermal keratinocytes and evaluated the positive in vivo response of mice engrafted with these genetically modified cells. Based on these studies, a similar approach exploited modified lentiviral and adenoviral vectors for the same purpose [152].

One of the natural consequences of this approach was the autologous transplantation of ex vivo genetically modified cells: Using a retrovirus-based strategy, Warrick et al. (2012) [152] transduced the functional XPC gene in undifferentiated human keratinocytes from one XPC patient and subsequently re-implanted.

The development of cell culture technologies made possible the assessment of in vitro-reconstructed skin, using genetically modified keratinocytes obtained from a skin biopsy of a XP patient. This strategy demonstrated to be efficient to generate an ex vivo full thickness skin that can be successfully engrafted on a resected area ensuring the protection from UV, without triggering the transplant-induced immune response [153].

To date, the gene therapy is considered by the scientific community as the elective treatment to counteract XP symptoms, even though it still requires technical implementations. Although positively validated in vivo, this strategy has showed potentially dangerous complications, such as uncontrolled random integrations and important side effects in the hematopoietic system [153,154].

In order to avoid the detrimental effects induced by the random incorporation of the gene of interest, the traditional technique has been implemented in order to allow a site-specific integration in non-coding regions of the host genome. This method is based on non-viral carriers, in particular on engineered site-specific endonucleases such as meganucleases or the most recent CRISPR/Cas9 [155,156,157]. In particular, Dupuy et al. (2013) [154] produced a meganuclease useful to selectively target the *XPC* gene. Despite its promising activity, this endonuclease demonstrated to be sensitive to epigenetic modifications such as DNA methylation, that limits its efficacy [154,158]. A similar but more elegant mechanism has been described for CRISPR/Cas9. CRISPR is a complex characterized by a sequence-specific ribonucleotide [CRISPR RNA (crRNA)] and by the transactivator crRNA (tracrRNA). This ribonucleoprotein complex, when coupled with the Cas9 endonuclease, induces the cleavage of DNA target through the recognition mediated by crRNA. To date, the CRISPR/Cas9 method has been successfully applied as gene therapy to numerous pathologies; its high versatility opens the possibility that this system could be also used to correct mutations underlying XP [125].

Gene therapy is a promising field of the personalised medicine because of its extreme plasticity, that is fundamental to counteract the gene heterogeneity of xeroderma pigmentosum. Nevertheless, despite the easiness of the above-described methods, the risk of off-target mutations and the modification of the human genome are still crucial issues for numerous scientists. Moreover, one common denominator of these methodologies is the necessity to perform a bioptic sampling to obtain autologous cell populations.

Another critical point related to the gene therapy consists in the timing of expression of the artificially introduced gene. It is reported that its expression rarely lasts more than two turnovers in ex vivo-produced skin [159]. Hence, multiple tissue engrafts or cell injections are needed to ensure continuity in therapy [160].

Currently, the highlighted technique-related limits induce most dermatologists to recommend alternative methods to negatively control the actinic keratosis in XP patients.

### 5.2. Liposomal Formulations as Nanocarriers for DNA Repair Enzymes

To target the high heterogeneity grade of mutations in XP, numerous personalized medicine approaches have been proposed. Among them, in the last two decades, the protein delivery mediated by liposomes is focusing great attention.

The application of lipidic nanoparticles as a therapeutic strategy has been widely expanded since their introduction in 1960s because of their high versatility. Due to their globular lipidic bilayered structure, liposomes have been recognized as one of the most promising drug delivery systems in numerous medical fields [161]. The large adaptability of this tool primarily derives from the wide variety of cargo therapeutic biomolecules that can be preserved and delivered to tissues/cells, such as both hydrophilic and hydrophobic drugs. Moreover, the suitability of liposomes as drug vehicles is due to their biocompatibility and biodegradability, while ensuring toxicity, non-immunogenicity, and low production costs [162]. To date, due to their benefits described above, liposomes are considered the most effective and safe drug-delivery system, thus often accepted as therapeutic option. 

Liposomes are commonly classified based on different parameters, that include the method for their production, the diameter and the lamellarity. Moreover, the encapsulation efficiency and the localization of the active principle in the liposomal membrane is strictly dependent on the their chemical/physical properties [163].

In comparison to the most of pharmacological options available for the treatment of several diseases, liposomes are efficient to ensure the protection of the carried molecule from the enzymatic degradation or immunologic/chemical inactivation. This drug defence avoids a premature activation or disruption of the active principle and, at the same time, minimizes the systemic absorption of the free-agent, thus increasing the therapeutic index and reducing the adverse effects [163,164,165]. Patil et al. (2016) [166] observed that anti-cancer drugs such as anthracyclines reduce their toxicity by the 50% when encapsulated in liposomes.

The drug delivery efficiency/timing is highly dependent on (i) the nature of phospholipids, that influence the interaction with the cell membrane; (ii) the nature/size of the encapsulated molecule; and (ii) the charge on the surface layer [165,167]. For instance, several studies demonstrated that anti-lymphoma compounds, when carried by liposomes, improve their efficacy because of a prolonged release/half-life [168,169,170].

Hence, the optimization of the physical/chemical properties of liposomes exerts a pivotal role to reach an optimal therapeutic activity, because of the direct proportionality between the release rate and the bioavailability of a pharmaceutical compound. In this regard, numerous chemical modifications (functionalization) have been developed and successfully tested to improve liposomal performances, such as immunogenicity reduction, enhanced half-life, specificity for a target tissue and sensitivity to environmental stimuli [171].

The rapid advances in the field of personalized medicine and biotechnologies led to the constant increase of the therapeutic compounds efficiently encapsulated in liposomes, in order to treat a wide range of diseases. Currently, more than 200 liposomal formulations containing proteins, peptides or nucleic acids (i.e., mRNA) have been approved by Food and Drug Administration (FDA) as strategies to treat a wide range of human diseases [172,173]. Among these, more than 60% are produced as anti-cancer therapy, such as the Doxil^®^ and DaunoXome^®^ (containing the doxorubicin and daunorubicin, respectively). Other preparations widely commercialized are anti-fungal (i.e., the AmBisome^®^, that vehicles the amphotericin B), liposomal vaccines (i.e., the Epaxal^®^, against the Hepatitis A virus and the most recent Comirnaty^®^, direct against the SARS-CoV-2), and also medical devices, such as Novasome^®^, which has been released as a dermo-cosmetic formulation [165,174,175].

This evidence supports the extreme versatility of lipid nanoparticles as an optimal medical device: in 2019, more than 70% of nanoparticles-based clinical trials proposed liposomes as therapeutics, vaccines, and diagnostics [165].

In the past, among the human diseases considered as an ideal target of therapeutic strategies based on nanoparticles, there was the xeroderma pigmentosum. The approach that most aroused scientists’ attention for the treatment of XP consists of a liposomal formulation that vehicles the T4 endonuclease 5 (T4N5), a DNA repair enzyme. The T4N5 is a pyrimidine-dimer-specific enzyme that is constitutively produced by the bacteriophage T4 [176,177]. It has been widely characterized because of its capability to catalyse the first step of NER in infected bacteria (i.e., *Escherichia coli*), through the disruption of the glycosylic bond and the consequent removal of the base of the 5’-pyrimidine within the dimer [178,179]. Tanaka et al. (1975) [180], based on its known mechanism of DNA repair, for the first time proposed the T4N5 to counteract UV-induced lesions in cells isolated from a XP patient. In order to efficiently vehicle the enzyme, the first proposal consisted in the infection of XP cells through the protein hemagglutinating virus of Japan (HVJ). A further study allowed to demonstrate that this approach results in the increased XP cell survival after UV exposure, together with the restoration of DNA damages to a normal level [178].

In order to avoid the possible toxic effect induced by the virus, further studies explored alternative delivery systems such as liposomes, demonstrating their in vitro ability to efficiently vehicle the T4N5 to human cells [181,182]. Further studies performed by Yarosh et al. (1991) [183] demonstrated that egg-derived liposomes are more efficient than other delivery systems to vehicle T4N5, allowing the use of lower concentrations of the enzyme. Moreover, this liposomal formulation was demonstrated to be efficient to trigger the activation of NER in both XP keratinocytes and in healthy keratinocytes/fibroblasts. First in vivo studies were performed in 1994 on a mouse model: the efficacy of T4N5 liposomal formulation was evaluated through its rate of absorption and its capability to modulate the activation of epidermal immune cells (i.e., Langerhans cells and macrophage infiltration) through the modulation of inflammatory mediators, such as Tumour Necrosis Factor (TNF) α [127,184,185,186].

Preliminary clinical studies using Dimericine (the commercial name of T4N5-liposomes) started in 1999, based on the strong evidence previously accumulated by Yarosh et al. (1999) [187]. Subsequently, in vivo studies performed on XP patients showed that the topical applied liposomal formulation containing T4N5 is able to penetrate the *stratum corneum* and to reach the dermis [39,115,188]. Recently, drugs encapsulated in liposomes are demonstrated to deeply penetrate into the skin and to generate a reservoir into the dermis [189]. Moreover, Dimericine is reported to be efficient to repair DNA damages also in melanocytes, with consequent increased melanogenesis [186].

In 2011, Camp et al. [190] evaluated the long-term response to the topical treatment using Dimericine. After 1 year of treatment, this medical device was able to reduce the incidence of new actinic keratoses and basal-cell carcinomas through an age-dependent effect, that resulted significant only in patients younger than 18 years old, without any immune response against T4N5.

To date, some clinical trials result as successfully completed: the efficacy of Dimericine was studied in XP patients, that were randomly assigned to receive T4N5 liposomes or a placebo lotion. The 1-year trial proved that T4N5 is efficient to reduce the rate of AK (−68%) and BCC onset in sun-damaged skin [191]. A further randomized placebo-controlled trial was performed in order to evaluate the anti-cancer activity of Dimericine in 100 immunosuppressed patients (underwent to kidney transplantation). The lotion was applied to sun-exposed areas of the skin (i.e., head, neck, face, and upper extremities) once daily for 12 months (ClinicalTrials.gov (accessed on 10 September 2021) Identifier: NCT00089180, status: completed) [191,192].

The successful accomplishment of these trials suggested that liposomes containing T4N5 could be an effective XP treatment, although continuing and careful use of the lotion throughout life is necessary [108,186,188]. Additionally, the phase III study of T4N5 liposomal topical lotion was FDA approved, but no data were collected to complete the clinical trial (ClinicalTrials.gov (accessed on 10 September 2021) Identifier: NCT00002811, status: unkown) [193].

Despite this, no evidence against the use of T4N5 liposomal formulations have been reported. In particular, acute and chronic safety tests have been performed in both mice and humans, demonstrating that no adverse reactions or significant alterations in skin biology are induced by Dimericine [84,194].

Due to their biocompatible characteristics, high versatility, extreme safety and low toxicity, T4N5-loaded liposomal formulations are proposed by dermatologists as a promising approach for the personalized treatment of XP and XP-related skin cancers. 

## 6. Conclusions and Future Perspectives

In summary, the topical treatment with Dimericine demonstrated in vitro and in vivo to be efficient to reduce CPDs and to modulate inflammatory mediators in UV-induced immunosuppression, without inducing any toxic effects. Nevertheless, some studies highlighted in animal models that Dimericine does not protect against melanoma development, probably due to the activation of molecular mechanisms independent from the accumulation of CPDs [195].

Notwithstanding their wide applications in clinic and research, liposomal formulations could promote a limited therapeutic outcome. One of the most frequent issues about the topical application is the short retention time after their administration. Thus, the composition of liposomes and the modification of their physical/chemical features have been modified to enhance their activity [196]. For instance, based on the target site, the size has been changed to improve their delivery efficacy [197]. Alternatively, in order to reach the optimal therapeutic half-life and stability, liposomes have been functionalized (i.e., polyethylene glycol (PEG)-ylated) [163]. Recently, the preparation methods have been also improved to guarantee a better control of their spatial/temporal biodistribution [163].

A substantial body of evidence accumulated over the past 20 years supports the concept that liposomal formulations containing T4N5 are a very promising topical treatment to prevent aging in healthy people [84] and actinic keratosis or NMSC in XP patients.

Clinical trials have demonstrated that Dimericine is highly safe and more efficient than other therapeutic approaches used by clinicians to control the development of skin cancer in high-risk populations, including XP patients. Dimericine is better accepted and tolerated by patients in comparison to other topical products. Moreover, its adverse effects have been shown to be almost abolished. 

However, the incomplete phase III clinical trial, together with accumulated data about the half-life and the spatial/temporal distribution of Dimericine underline the need of further improvements of this medical device. A liposomal formulation with optimized loading efficiency, retention and stability could be essential to promote a larger use of T4N5-based approach in dermatology.

## Data Availability

Not applicable.
